# Weight loss in the healthy elderly might be a non-cognitive sign of preclinical Alzheimer's disease

**DOI:** 10.18632/oncotarget.22218

**Published:** 2017-10-31

**Authors:** Amanda Jimenez, Jordi Pegueroles, María Carmona-Iragui, Eduard Vilaplana, Victor Montal, Daniel Alcolea, Laura Videla, Ignacio Illán-Gala, Adriana Pané, Anna Casajoana, Olivia Belbin, Jordi Clarimón, Violeta Moizé, Josep Vidal, Alberto Lleó, Juan Fortea, Rafael Blesa, The Alzheimer's Disease Neuroimaging Initiative

**Affiliations:** ^1^ Endocrinology and Diabetes Department, Obesity Unit, Hospital Clinic de Barcelona, Barcelona, Spain; ^2^ Institut d'Investigacions Biomèdiques August Pi Sunyer, Barcelona, Spain; ^3^ Memory Unit, Department of Neurology, Hospital de la Santa Creu i Sant Pau, Biomedical Research Institute Sant Pau, Universitat Autònoma de Barcelona, Barcelona, Spain; ^4^ Centro de Investigación Biomédica en Red de Enfermedades Neurodegenerativas (CIBERNED), San Sebastian, Spain; ^5^ Barcelona Down Medical Center, Fundació Catalana de Síndrome de Down, Barcelona, Spain; ^6^ General Surgery Service, Hospital de Barcelona-SCIAS, Barcelona, Spain; ^7^ Centro de Investigación Biomédica en Red de Diabetes y Enfermedades Metabólicas Asociadas (CIBERDEM), Barcelona, Spain

**Keywords:** pre-clinical Alzheimer’s disease, weight loss, cerebrospinal fluid Alzheimer’s disease biomarkers, PET amyloid, magnetic resonance imaging, Gerotarget

## Abstract

Weight loss has been proposed as a sign of pre-clinical Alzheimer Disease (AD). To test this hypothesis, we have evaluated the association between longitudinal changes in weight trajectories, cognitive performance, AD biomarker profiles and brain structure in 363 healthy controls from the Alzheimer´s Disease Neuroimaging Initiative (mean follow-up 50.5±30.5 months). Subjects were classified according to body weight trajectory into a weight loss group (WLG; relative weight loss ≥ 5%) and a non-weight loss group (non-WLG; relative weight loss < 5%). Linear mixed effects models were used to estimate the effect of body weight changes on ADAS-Cognitive score across time. Baseline CSF tau/AΔ_42_ ratio and AV45 PET uptake were compared between WLG and non-WLG by analysis of covariance. Atrophy maps were compared between groups at baseline and longitudinally at a 2-year follow-up using Freesurfer. WLG showed increased baseline levels of cerebrospinal fluid tau/AΔ_42_ ratio, increased PET amyloid uptake and diminished cortical thickness at baseline. WLG also showed faster cognitive decline and faster longitudinal atrophy. Our data support weight loss as a non-cognitive manifestation of pre-clinical AD.

## INTRODUCTION

Unintentional weight loss in elderly subjects has been related to disability and increased mortality [1, 2]. Dementia is a major cause of disability and mortality in the elderly, and has also been associated with weight loss [3–5]. Moreover, dementia severity correlates with weight loss and malnutrition [3]. This weight loss in the context of dementia could be explained by a lower energy intake due to significant neuropsychological symptoms related to disease progression.

Several epidemiological studies, nonetheless, have also identified weight loss preceding the onset of dementia [6–11], and recently published epidemiological data have consistently shown that weight loss may precede mild cognitive impairment (MCI) [7, 12, 13]. This last observation suggests that weight loss could be a non-cognitive sign of preclinical Alzheimer's Disease (AD). According to NIA-AA criteria, preclinical AD is defined by evidence of brain amyloidosis through biomarkers [14] (labeled as asymptomatic at-risk in the IWG lexicon [15]). However, there is a lack of published multimodal studies assessing the relationship between weight loss, cognition, AD biomarkers and brain structure in the healthy elderly.

We hypothesized that weight loss in elderly subjects is a non-cognitive sign of preclinical AD that could predict cognitive decline and would be associated with brain atrophy and AD biomarkers. In this longitudinal study, we aimed to examine (1) changes on cognitive performance over time, (2) the baseline AD biomarker profile and (3) cross-sectional and longitudinal brain structural changes according to body weight trajectories in healthy controls from the Alzheimer´s Disease Neuroimaging Initiative (ADNI).

## RESULTS

**Figure 1:**
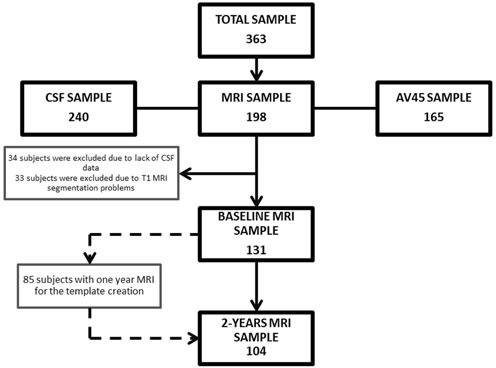
Flowchart showing the samples used in this work and the subsets utilized for analyses

Three hundred and sixty three subjects were included in the study (Figure 1). The mean age was 74.9±5.8 years, 189 were female, and the mean baseline BMI was 26.9±4.0 Kg/m^2^. At a mean follow-up of 50.5±30.5 months (range: 12 to 120 months), the mean weight change for the whole cohort was −2.2±6.3% of baseline body weight (range: −23.1% to +19.1%). Eighty nine of the 363 subjects (24.5%) lost ≥5% of total body weight. Table 1 summarizes the demographic, anthropometric and neuropsychological data at baseline according to the weight loss trajectory. The WLG had a slightly higher baseline BMI than the non-WLG. No other significant differences were found at baseline evaluation between both groups. Follow-up was longer in the WLG than in the non-WLG group (45.0±28.0 months *vs*. 64.2±33.1 months, *p* < 0.001).

**Table 1:** Demographic, anthropometric and neuropsychological data at baseline according to the weight trajectories for whole cohort and PET-AV45, CSF and MRI subsets

	Whole cohort (n=363)		PET AV45 sample (n=165)		CSF sample (n=240)		Cross MRI sample (n=131)		Long MRI sample (n=104)	
	WLG (n=89)	n-WLG (n=274)	WLG (n=29)	n-WLG (n=136)	WLG (n=58)	n-WLG (n=182)	WLG (n=31)	n-WLG (n=100)	WLG (n=23)	n-WLG (n=81)
Age, mean (SEM), years	75.61 (5.82)	74.62 (5.80)	73.83 (6.94)	73.47 (6.19)	74.73 (6.21)	74.58 (6.10)	74.13 (6.58)	73.37 (6.19)	74.86 (6.71)	73.53 (6.24)
Gender [F, (%)]	43 (48.3)	146 (53.3)	13 (44.8)	72 (52.9)	27 (46.6)	95 (52.2)	12 (38.7)	55 (55.0)	9 (39.1)	47 (58.0)
BMI, mean (SEM), Kg/m^2^	27.72 (3.83)	26.56 (4.14)*	27.72 (3.59)	27.21 (4.07)	27.64 (3.89)	26.58 (3.99)	25.5 (4.0)	27.1 (4.0)	27.82 (3.87)	26.96 (3.59)
SBP, mean (SEM), mmHg	134.85 (16.28)	134.18 (16.02)	137.59 (16.60)	134.32 (15.89)	133.95 (16.15)	133.45 (16.02)	138.32 (13.94)	131.51 (15.52)	139.35 (13.00)	132.53 (14.60)
DBP, mean (SEM), mmHg	74.61 (9.04)	74.55 (10.30)	74.48 (9.92)	74.72 (9.98)	74.14 (9.33)	74.83 (10.44)	72.58 (8.63)	74.44 (10.98)	72.48 (8.88)	73.98 (10.54)
FPG, mean (SEM), mg/dL	101.25 (18.50)	99.38 (19.91)	92.14 (12.93)	99.17 (18.08)	99.66 (18.64)	99.22 (18.91)	97.26 (16.55)	98.83 (19.27)	96.70 (15.15)	98.12 (14.78)
Cholesterol, mean (SEM), mg/dL	190.97 (39.14)	191.88 (39.11)	184.48 (35.76)	191.49 (37.25)	190.19 (38.07)	191.00 (38.60)	184.68 (31.02)	189.21 (38.88)	183.04 (32.13)	188.35 (40.74)
Triglicerydes, mean (SEM), mg/dL	159.00 (100.31)	134.88 (76.70)	138.79 (95.08)	135.66 (73.83)	161.41 (101.57)	131.92 (76.97)	126.77 (86.99)	138.11 (71.10)	136.35 (97.22)	139.46 (76.18)
T2D [n, (%)]	15 (16.9)	27 ( 9.9)	2 ( 6.9)	10 ( 7.4)	10 (17.2)	17 ( 9.3)	3 ( 9.7)	8 ( 8.0)	2 (8.7)	5 (6.4)
Education, mean (SEM), years	16.15 (2.79)	16.41 (2.67)	16.55 (1.99)	16.55 (2.70)	16.28 (2.66)	16.19 (2.73)	16.19 (2.20)	16.55 (2.49)	15.96 (1.82)	16.63 (2.52)
ADAS-Cog score, mean (SEM)	8.87 (4.24)	9.39 (4.39)	9.17 (4.30)	9.14 (4.56)	9.00 (4.16)	9.36 (4.55)	8.35 (3.92)	8.82 (4.68)	8.42 (3.96)	8.75 (4.59)
APOE4-carrier [n, (%)]	27 (30.3)	80 (29.2)	7 (24.1)	43 (31.6)	15 (25.9)	49 (26.9)	8 (25.8)	32 (32.0)	6 (26.1)	25 (30.9)

WLG: weight loss group; non-WLG: non weight loss group. CSF: cerebrospinal fluid; MRI: magnetic resonance image; BMI: body mass index, SBP: systolic blood pressure, DBP: diastolic blood pressure, FPG: fasting plasmatic glucose, T2D: type 2 diabetes, MMSE: minimental score examination, ADAS-Cog: Alzheimer's Disease Scale Assessment.,*: p<0.05,

### Weight loss is associated with faster cognitive decline

A total of 2,051 observations were used in the linear mixed effects analysis including follow-up of the 363 subjects. Linear mixed effects analyses showed a significant interaction between time and body weight changes on the ADAS-Cog both when analyzing weight changes as categorical and when analyzing weight changes as a continuous variable. WLG showed a mean (SEM) increase of 0.57 (0.15) ADAS-Cog points more per year than the non-WLG (*p* < 0.001). Higher relative weight loss was also associated with a faster cognitive decline (*p* < 0.01) when treated continuously (Table 2). The inclusion of baseline BMI or T2D status as covariates did not change the results.

**Table 2:** Multivariable Mixed-Effects Linear Models for Alzheimer's Disease Scale Assessment scores

Effect	Mean estimate^a^ (SE)	p-value
**Model 1 (dependent variable: ADAS-Cog score (number of observations: 2051 )**		
Weight variation (% of baseline)	0.043 (0.033)	0.194
Weight variation (% of baseline)*time (months)	−0.030 (0.009)	<0.001
**Model 2 (dependent variable: ADAS-Cog score (number of observations: 2051 )**		
Weight-loss group	−0.689 (0.449)	0.125
Weight-loss group*time (years)	0.575 (0.150)	<0.001

ADAS-Cog: Alzheimer's Disease Scale Assessment scores. a: Adjusted for age, sex and time.

**Figure 2:**
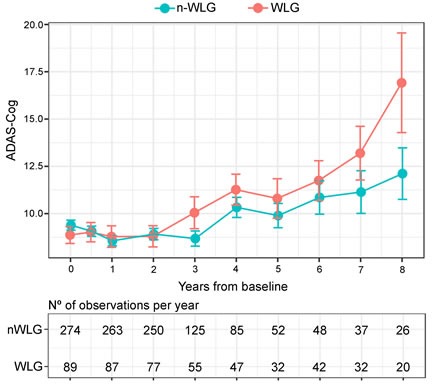
Observed means (SEM) differences in Alzheimer's Disease Scale Assessment Scores according to body weight trajectory groups. In blue is represented the ADAS-Cog trajectory for the non-WLG throughout time and in red is represented the ADAS-Cog trajectory for the WLG throughout time. Dots represent mean of each time-point for both groups and the error bars show the standard error of each time-point for both groups. In the lower panel it is shown the number of observation per year.

Figure 2 displays the unadjusted mean ADAS-Cog score and their corresponding standard error of mean according to the WL categories at each follow-up visit.

### Weight loss is associated with AD biomarkers in the healthy elderly

Two hundred and seventy three study participants had available baseline CSF or AV45 PET data. Subjects with baseline CSF or AV45 PET data were younger than the rest of participants (74.4±6.1 *vs*. 76.1±4.9 years, *p* = 0.006). No other significant differences were found between this subset and the whole cohort (Supplementary Table 1).

Demographic, anthropometric and neuropsychological data at baseline according to weight trajectories in the AD-biomarkers subset are presented in Table 1. No significant differences between WLG and non-WLG were found at baseline evaluation.

In the ANCOVA analysis, after adjustment for age, sex and *APOE* genotype, WLG showed higher baseline AV45 PET SUVR (1.179±0.248 *vs*. 1.098±0.159; *p* = 0.020) and higher CSF total tau/AΔ_42_ ratio (0.454± 0.319 *vs*. 0.355±0.224, *p* = 0.005) compared to non-WLG. Inclusion of baseline BMI or T2D status as covariates did not significantly change the association between weight trajectories and CSF total tau/AΔ_42_ ratio (*p* = 0.005 and *p* = 0.005, respectively) or between weight trajectories and AV45 PET SUVR (*p* = 0.020 and *p* = 0.019, respectively).

Exclusion of subjects that progressed to MCI or dementia in the follow-up (*n* = 49) did not change association between weight loss and total tau/AΔ_42_ ratio (0.33± 0.21 *vs*. 0.44±0.32, *p* = 0.01) or between weight loss and AV45 PET SUVR (1.091±0.156 *vs*. 1.182±0.252; *p* = 0.016).

In the linear regression analyses we observed similar results. There was a trend for a positive correlation between weight loss levels and baseline t-tau/AΔ_42_ ratio (*p* = 0.077) and a significant association between weight loss levels and baseline AV45 uptake (*p* = 0.022).

### Weight loss is associated with baseline cortical thinning and accelerated atrophy rates

Table 1 shows the clinical characteristics of the study participants with available MRI at baseline and 2-years follow-up. There were no significant differences between this subset (nor the longitudinal MRI subset) and the whole cohort (Supplementary Table 2).

**Figure 3:**
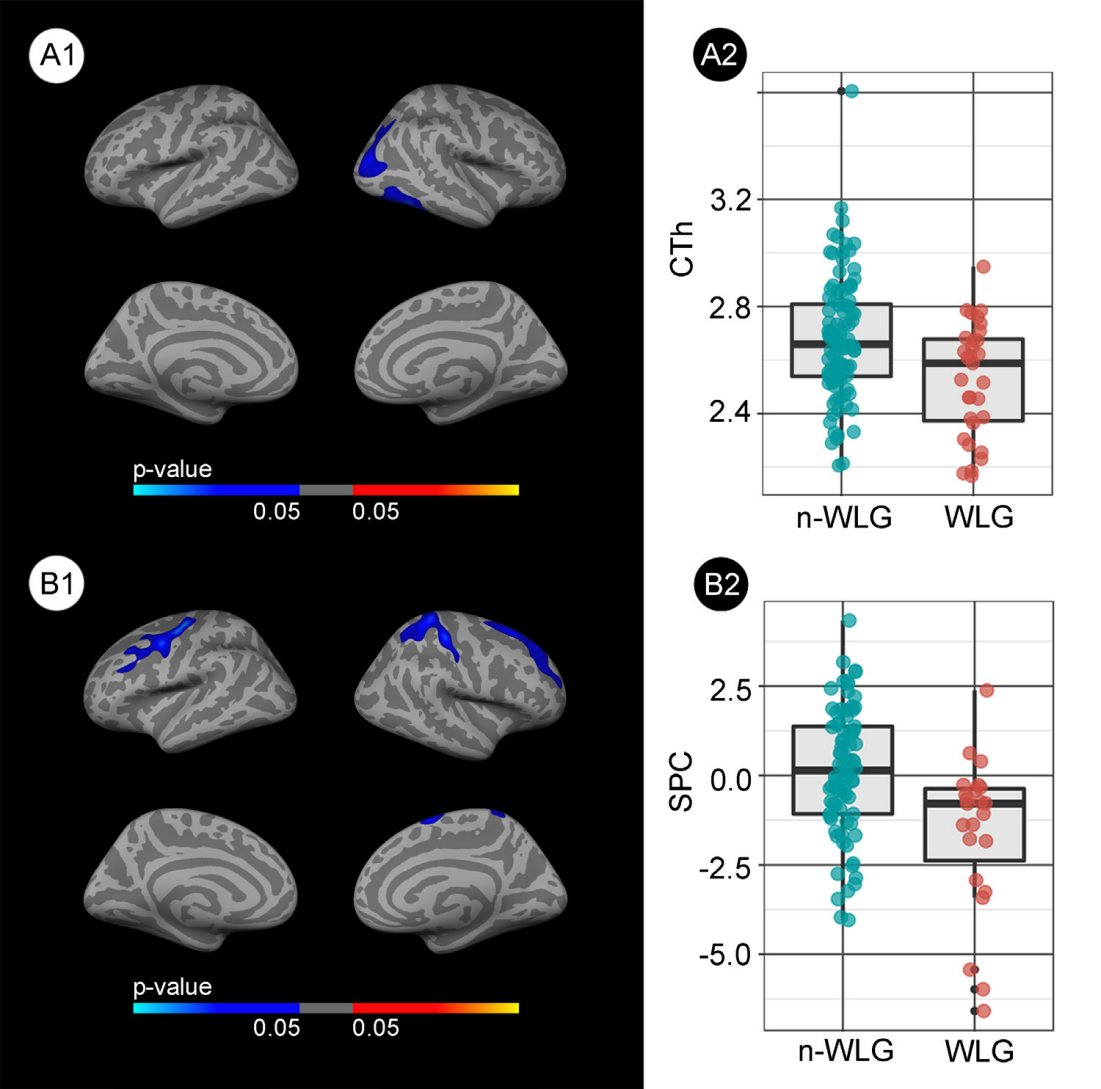
Group comparison of the structural changes between the weight loss group (WLG) and non-weight loss group (non-WLG) **A1.** Baseline differences in cortical thickness between the weight loss group (WLG) and the non-weight loss group (non- WLG). Blue areas represent significant cortical thinning in the WLG respect to the non-WLG (FWE < 0.05). **A2.** Box and whisker plots showing the cortical thickness for each group at the maximum significant vertex in the right temporal cluster. **B1.** Differences in the symmetrized percent change (spc) over the 2-year follow-up between WLG and non-WLG. Blue indicates significant increased cortical atrophy (i.e., less spc) in the WLG (FWE < 0.05). **B2.** Box and whisker plots showing the symmetrized percent change for each group at the maximum significant vertex in the right frontal cluster. The colors in the box-plots are only for illustrative purposes.

MRI results are presented in Figure 3. At baseline, the WLG presented cortical thinning in temporal regions of the right hemisphere (Figure 3A) with respect to the non-WLG. Subjects in the WLG also showed accelerated atrophy rates with respect to the non- WLG in widespread areas of both hemispheres (Figure 3B). Neither adjustment for AD biomarkers nor inclusion of T2D or baseline BMI as covariates significantly altered the results (data not shown). The boxplots illustrate the median CTh and spc for each group and the respective upper and lower quartiles (Figures 3A2 and 3B2).

No significant differences were found in CTh between the WLG and the non-WLG when subjects that progressed to MCI or dementia were exluded. In the longitudinal analysis, the WLG showed several clusters of accelerated cortical thinning in the precuneus and frontal superior areas of both hemispheres (FWE *p* < 0.05) when compared to the non-WLG (Supplementary Figure 1).

In the correlation analyses weight loss was associated with cortical thinning in the same areas identified in the group comparison analyses. No significant correlations between relative weight change and spc were found (Supplementary Figure 2).

## DISCUSSION

Here we report the first published study to examine the influence of weight loss on cognitive decline and on AD CSF and imaging biomarkers in a large multicenter cohort of cognitively normal individuals. Our data support the hypothesis that unintentional weight loss in healthy elderly subjects is a non-cognitive sign of preclinical AD and demonstrate a significant association between weight loss and cognitive decline in healthy elderly subjects. Specifically, those subjects with significant weight loss presented an extra 0.57 points per year in ADAS-Cog score compared to those participants without weight loss. These data are in agreement with previously published epidemiological studies identifying weight-loss as a risk factor for incident MCI [7, 12, 13].

In contrast to a previous study [26], we also report an association between weight loss and increased AD biomarker levels. That being said, this discrepancy may be due to the inclusion of individuals with MCI and dementia in the previous study and that the BMI change was assessed as a continuous variable [26]. As mentioned in the methods section, this approach could have reduced sensitivity for detecting an association between weight loss and biomarker levels. Moreover, the previous study included fewer HC than this study. Our data are, however, in agreement with a previous autopsy study which found that AD plaque and tangle pathology was related to declining BMI in the years preceding death in demented and non-demented subjects [27].

We also describe an association between weight loss and baseline and longitudinal atrophy. Admittedly, in addition to AD-vulnerable areas, some of the clusters were found in areas atypical for AD. Therefore, we cannot exclude that, at least in part, other non-AD related neurodegenerative processes might contribute to weight loss and cognitive decline. This association between body weight changes and brain structure in HC has seldom been reported [28, 29]. Driscoll et al. in a large cohort of healthy elderly women found that a significant BMI decline (also defined as >5% of BMI from baseline to the last visit) in the years preceding the MRI scan (mean of 6.6 years) was cross-sectionally associated with reduced temporal grey matter volume and reduced volume of the cingulate cortex and hippocampus [29]. Similarly, in a longitudinal study, Bobb et al., found that weight loss over a five-year period was significantly associated with a decline in hippocampal volume, but this study did not find changes in the cortical mantle [28]. It may be pertinent that the mean age in that study was significantly younger than in the present study (60.1 *vs*. 74.9 years*).*

Finally, it is noteworthy that the association between weight loss, AD biomarkers and brain atrophy in healthy ADNI participants remained significant even after the exclusion of subjects with progression to MCI or dementia in the follow-up, suggesting that weight loss starts early in preclinical AD stages.

The underlying mechanism that links weight loss to preclinical AD has yet to be elucidated but could be attributed to many factors. First, symptoms such as depression or anxiety, frequently observed during the prodromal (and preclinical) stages of AD, may affect appetite and energy intake. That being said, previous epidemiological studies show that the association between weight loss and incident dementia remains significant even after controlling for depressive symptomatology [30, 31]. Second, olfactory impairment has been observed in subjects with MCI and dementia and has been related to decreased hippocampus volume, thinner entorhinal cortex and greater amyloid burden in normal cognitive subjects [32]. Moreover, a recent study identified olfactory dysfunction as a predictor of amnestic MCI [33], suggesting that impaired olfaction is present very early in the AD continuum. Impaired olfaction may alter gustatory sensitivity and may thus influence food preferences and contribute to reduced calorie intake. Interestingly, previous studies have identified diminished gustatory sensitivity in both MCI and AD patients [34, 35]. Third, AD might affect the neuronal circuits involved in the regulation of eating behavior causing significant alterations in energy intake. Although homeostatic regulation of energy intake is located in the hypothalamus and brainstem, this homeostatic system is strongly influenced and could be overridden by hedonic signals. Hedonic eating is governed by the brain reward system, and the brain regions responsible of the reward system are dispersed in the corticolimbic structures [36]. In this respect, in animal studies the injection of β-amyloid into the hipoccampus caused reduced energy intake and decreased body weight [37, 38]. Finally, AD has also been associated to a hypermetabolic state which could explain the relationship with weight loss. In animal models overexpressing the APP gene, an increased energy expenditure without alterations in feeding behavior has been observed prior to plaque formation [39]. In humans, nonetheless, previous studies evaluating the total energy expenditure and its components (resting energy metabolism, diet induced thermogenesis and physical activity energy expenditure) did not reveal significant differences between subjects with or without AD dementia [40]; however energy expenditure has yet to be assessed in preclinical AD subjects. It is possible that these proposed mechanisms could occur individually or in combination and may change over the decades-long AD process.

Our results may have direct implications for clinical practice. First, unexplained weight loss in the healthy elderly could help to identify persons at-risk for MCI and AD dementia. Second, these data support the need for a careful nutritional evaluation in subjects with incident MCI or dementia, as poor nutritional status, loss of muscle mass and specific micronutrients deficits can be expected after a long period of negative energy balance. Finally, these results may be of relevance when assessing the relation between late-life obesity, AD biomarkers and AD risk and might help to explain the so called “obesity paradox”. Thus, although mid-life obesity has been consistently linked to AD and AD pathology [41], the relationship between late-life obesity and dementia is controversial [42]. Weight loss could be a confounding factor in this association. Further studies describing weight trajectories across the AD continuum are needed to better define the precise temporal association between preclinical AD and body weight changes.

Several limitations should be considered when interpreting the data reported here. Firstly, intentional weight loss cannot be differentiated from unintentional weight loss in the ADNI cohort. However, since previous studies have shown that intentional weight loss in healthy obese subjects and in obese MCI patients has beneficial cognitive effects [43–45], it is unlikely that the observed weight loss in our study was intentional. A further limitation is that we cannot specify the type of body composition changes or quantify physical activity levels [46, 47] since this information is not available in ADNI database. Third, subjects with significant weight loss had longer follow-up than subjects without weight loss. Nonetheless, differences were found in all biomarkers at baseline and the statistical methods employed were robust to differences in follow-up across groups.

In conclusion, our data support that weight loss in healthy elderly is a non-cognitive sign of preclinical AD. Weight loss is related to faster cognitive decline, accelerated brain atrophy rates and is correlated with AD biomarker. The mechanisms involved in this association warrant further investigation.

## MATERIALS AND METHODS

### Participants

Data used in the preparation of this article were obtained from the ADNI database (adni.loni.usc.edu). The ADNI was launched in 2003 by the National Institute on Aging (NIA), the National Institute of Biomedical Imaging and Bioengineering (NIBIB), the Food and Drug Administration (FDA), private pharmaceutical companies and non-profit

organizations, as a $60 million, 5-year public-private partnership. The primary goal of ADNI has been to test whether serial magnetic resonance imaging (MRI), positron emission tomography (PET), other biological markers, and clinical and neuropsychological assessment can be combined to measure the progression of mild cognitive impairment (MCI) and early AD. Determination of sensitive and specific markers of very early AD progression is intended to aid researchers and clinicians to develop new treatments and monitor their effectiveness, as well as lessen the time and costs of clinical trials.

The Principal Investigator of this initiative is Michael W. Weiner, MD, VA Medical Center and University of California – San Francisco. ADNI is the result of efforts of many co-investigators from a broad range of academic institutions and private corporations, and subjects have been recruited from over 50 sites across the U.S. and Canada. The initial goal of ADNI was to recruit 800 subjects, which has since been broadened to include ADNI-GO and ADNI-2. To date these three protocols have recruited over 1500 adults, ages 55 to 90, to participate in the research, consisting of cognitively normal older individuals, people with early or late MCI, and people with early AD. The follow-up duration of each group is specified in the protocols for ADNI-1, ADNI-2 and ADNI-GO. Subjects originally recruited for ADNI-1 and ADNI-GO had the option to be followed in ADNI-2. For up-to-date information, seehttp://adni-info.org/. We selected all cognitively healthy controls (HC) with available baseline and longitudinal anthropometric and cognitive data for a minimum follow-up period of 12 months. After revision of the ADNI subjects table, and in order to exclude other causes associated with weight loss, we excluded subjects that had: cancer, heart failure, renal disease, liver disease, stroke, gastroesophageal reflux disease, Lyme disease, pulmonary disease or Barretts disease. There were no differences between ADNI-GO and ADNI2 with respect to the healthy controls inclusion criteria, the protocols of clinical follow-up, CSF measure, FBP PET or structural MRI acquisitions.

### Clinical and cognitive data

Baseline demographic data (age, gender, education and Type 2 Diabetes (T2D) medication), neuropsychological performance, anthropometric data (height, weight, systolic and diastolic blood pressure) and laboratory data (fasting plasmatic glucose -FPG-, cholesterol, and triglycerides) were downloaded from the ADNI database. Diabetes status was assigned according to FPG ≥126 mg/dL (*n* = 20) and/or the use of glucose lowering agents (*n* = 22) [16]. Follow-up visits were conducted yearly. Data on body weight and ADAS-Cog score were downloaded from each subject at each follow-up visit. We selected ADAS-Cog score 13 as measure of cognitive performance because it is the most widely used general cognitive measure in clinical trials of AD [17].

### Assessment of body weight changes

Body weight change was expressed as relative weight change with respect to the weight at baseline [(follow-up weight – baseline weight) / baseline weight*100]. Relative body weight change was selected rather than total weight or BMI change in order to take into account baseline differences in body weight. Small changes of body weight can be due to measurement error, clothing, food consumption or fluid balance and may have little clinical relevance [18]. Therefore, following consensus criteria for wasting disease and current definition of meaningful weight loss [18, 19] the sample was categorized into two weight-change categories in the main analyses: Participants with relative weight loss ≥ 5% at last available follow-up were classified as weight loss group (WLG) whereas subjects with relative weight loss < 5% were classified as non-weight loss group (non-WLG).

### Genetic and CSF data

*APOE* genotype was determined at the ADNI Biomarker Core Laboratory. Participants were considered *APOE* ε4 positive (APOE4) if they carried at least one *APOE* ε4 allele. CSF biomarker values were obtained from the ADNI database. Two hundred and forty of 363 participants (66.1%) included in the study had CSF available at baseline. Methods for CSF acquisition and analysis have been previously reported [20]. We used the ratio between t-tau and AΔ_42_ (tau/AΔ_42_ ratio) to evidence the AD pathophysiological process[15]. T-tau was used instead of p-tau because in ADNI, t-tau has a higher specificity than p-tau (92,3% *vs* 73,1%) [20].

### Amyloid PET acquisition and processing

One hundred and sixty five subjects of 363 (45.5%) had available AV45 PET scan at baseline. The details for AV45 PET scan acquisition are available elsewhere (http://www.adni-info.org). Composite AV45 standardized uptake values ratios (SUVR) relative to the whole cerebellum were obtained from the ADNI database.

### MRI acquisition and analysis

We selected those participants with available 3T MRI. The details of MRI acquisition and pre-processing are available elsewhere (http://adni-info.org/). All structural MRIs were first processed using the cross-sectional cortical reconstruction stream in Freesurfer (v5.1; http://surfer.nmr.mgh.harvard.edu)[21, 22]. For the cross-sectional analyses, after excluding subjects with lack of CSF data (*n* = 34) and segmentation errors (*n* = 33), 131 baseline MRI were included. Cortical thickness was calculated as the distance from the grey/white matter boundary closest to the grey/CSF boundary at each vertex.

The longitudinal analyses were performed with the Freesurfer longitudinal stream [23]. Specifically, an unbiased within-subject template space and image is created using all time-point MRIs available, including 1-year follow-up (*n* = 57), for each subject [24]. After the exclusion of subjects with lack of baseline CSF data (*n* = 17), 104 were included in the longitudinal analyses. Symmetrized percent change (spc) values between baseline and two-year MRIs time-points were automatically extracted and introduced in the two stage model as implemented in Freesurfer [25]. Briefly, the spc measure is the annual rate of atrophy between two time-points with respect to the average thickness between them. Finally, both measures (cortical thickness and spc maps) were smoothed using a Gaussian kernel of 15mm full-width at half maximum.

### Statistical analysis

Demographic, anthropometric and cognitive variables were compared across groups using student t-tests, and χ^2^ tests. Linear mixed-effects models using the Maximum Likelihood method for the change in the ADAS-Cog score were performed on the entire longitudinal database. In the primary analyses, weight variation was treated as a dichotomous variable (WLG and non-WLG). Intercept, age, sex, weight change-category, time from baseline and the interaction between time and weight change-category were included as covariates. Intercept and time from baseline were included as subject-specific random effects. In the secondary analyses, weight changes were treated as a continuous variable (percentage with respect to baseline weight). Less than 10% of the cohort had a follow-up beyond 96 months. Therefore, the clinical analyses were censured at month 96. The sample size for each group and the observed mean (SEM) differences in ADAS-Cog13 at each visit were reported for each WL trajectory group.

The associations between weight changes with both individual baseline CSF biomarkers and AV45-SUVR were tested both using analysis of covariance –ANCOVA– (primary analysis) and regression analyses (secondary analysis). Statistical significance was set at 5% (alpha level = 0.05). R software (version 3.2.5, www.r-project.org) and *lme4* were used to perform the statistical analyses.

To study the association of CTh and spc with weight trajectories, group comparisons were performed between the WLG and the non-WLG (primary analyses) and correlation analyses (secondary analyses) using the QDEC tool in Freesurfer. We tested Monte-Carlo simulation with 10,000 repeats as implemented in Qdec (family wise error [FWE] correction at *p* < 0.05). The figures show only those results that survived FWE correction.

## SUPPLEMENTARY MATERIALS FIGURES AND TABLES


