# Supplementary material for: Weight loss in the healthy elderly might be a non-cognitive sign of preclinical Alzheimer's disease
Source: Oncotarget. 2017 Oct 31;8(62):104706–16. doi: 10.18632/oncotarget.22218 (PMC5739594; doi:10.18632/oncotarget.22218)
Supplement: Supplementary file 1 [file oncotarget-08-104706-s001.pdf]

Weight loss in the healthy elderly might be a non-cognitive sign of preclinical Alzheimer's disease

SUPPLEMENTARY MATERIALS

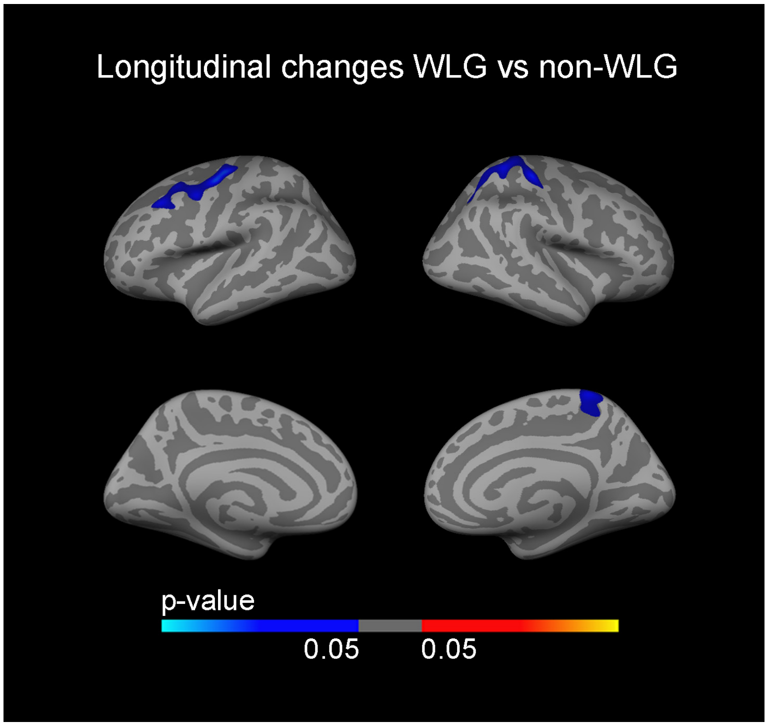

Supplementary Figure 1:

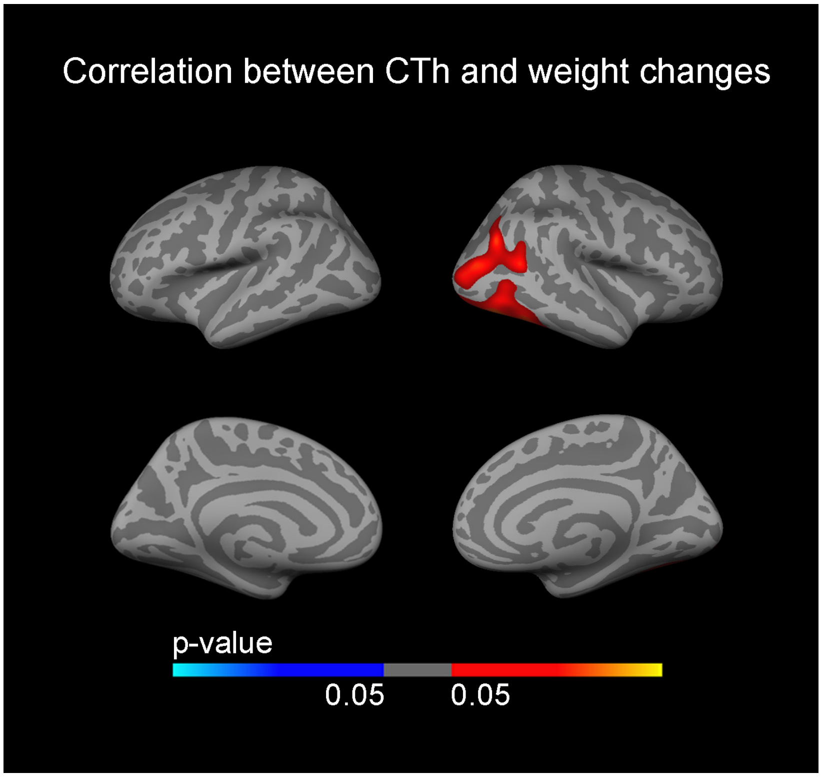

Supplementary Figure 2:

Supplementary Table 1: Clinical, neuropsychological and biochemical comparison between subjects with and without data on CSF Alzheimer's disease biomarkers or AV45 PET scan

	Subjects without CSF or AV45 data (n=97)	Subjects with CSF or AV45 data (n=266)	
Age (years), mean (SEM)	76.1 (4.9)	74.4 (6.1)	0.006
Gender (F) (%)	54 (55.7)	135 (50.8)	0.407
BMI (Kg/m^2), mean (SEM)	26.5 (4.2)	27.0 (4.0)	0.288
SBP (mmHg) mean(SEM)	135.5 (16.2)	133.9 (16.0)	0.398
DBP(mmHg), mean (SEM)	74.7 (9.9)	74.5 (10.0)	0.859
FPG (mg/dL), mean (SEM)	101.8 (22.1)	99.1 (18.5)	0.245
Cholesterol (mg/dL), mean (SEM)	192.2 (41.8)	191.4 (38.0)	0.876
Triglycerides (mg/dL), median (IQR)	123.0 (86.5-172.0)	114.5 (85.0-171.0)	0.314
T2D [n, (%)]	14 (14.4)	28 (10.5)	0.303
Education, mean (SEM)	16.6 (2.6)	16.3 (2.7)	0.336
ADAS-Cog score, mean (SEM)	9.1 (4.3)	9.3 (4.4)	0.694
ApoE4-carrier [n, (%)]	32 (33.0)	75 (28.2)	0.303

BMI: body mass index; F: females; SBP: systolic blood pressure; DBP: diastolic blood pressure, FPG: fasting plasmatic glucose; T2D: type 2 diabetes, ADAS-Cog score: Alzheimer's Disease Scale Assessment. SEM: standard error of the mean; IQR: interquartilic range; CSF: cerebrospinal fluid;

Supplementary Table 2: Clinical, neuropsychological and biochemical comparison between subjects with and without MRI data

	Subjects without 3T MRI scan (n=232)	Subjects with 3T MRI scan (n=131)	
Age (years), mean (SEM)	75.6 (5.4)	73.5 (6.3)	0.002
Gender (F) (%)	122 (52.6)	67 (51.1)	0.792
BMI (Kg/m^2), mean (SEM)	26.7 (4.1)	27.2 (3.9)	0.269
SBP (mmHg) mean(SEM)	135.0 (16.4)	133.1 (15.4)	0.277
DBP(mmHg), mean (SEM)	74.9 (9.7)	74.0 (10.5)	0.417
FPG (mg/dL), mean (SEM)	100.6 (20.1)	98.5 (18.6)	0.313
Cholesterol (mg/dL), mean (SEM)	193.6 (39.9)	188.1 (37.1)	0.199
Triglycerides (mg/dL), median (IQR)	120.0 (85.0-171.0)	111.0 (85.0-174.0)	0.595
T2D [n, (%)]	31 (13.4)	11 (8.4)	0.156
Education, mean (SEM)	16.3 (2.8)	16.5 (2.4)	0.511
ADAS-Cog score, mean (SEM)	9.6 (4.2)	8.7 (4.5)	0.068
ApoE4-carrier [n, (%)]	67 (28.9)	40 (30.5)	0.740

BMI: body mass index; F: females; SBP: systolic blood pressure; DBP: diastolic blood pressure, FPG: fasting plasmatic glucose; T2D: type 2 diabetes, ADAS-Cog score: Alzheimer's Disease Scale Assessment. SEM: standard error of the mean; IQR: interquartilic range.
